# Should additional domains be added to the EQ-5D health-related quality of life instrument for community-based studies? An analytical descriptive study

**DOI:** 10.1186/s12963-015-0046-0

**Published:** 2015-06-02

**Authors:** Jennifer Jelsma, Soraya Maart

**Affiliations:** Division of Physiotherapy, Department of Health and Rehabilitation Sciences, Faculty of Health Sciences, University of Cape Town, Anzio Road, Observatory, Cape Town, South Africa

**Keywords:** Health related quality of life, EQ-5D-3L, WHOQOL-BREF, Functional domains, Community survey

## Abstract

**Background:**

There is increasing interest in monitoring the health-related quality of life (HRQoL) of populations as opposed to clinical populations. The EQ-5D identifies five domains as being most able to capture the HRQoL construct. The question arises as to whether these domains are adequate within a community-based population or whether additional domains would add to the explanatory power of the instrument.

**Methods:**

As part of a community-based survey, the responses of 310 informants who reported at least one problem in one domain filled in the EQ-5D three-level version and the WHOQOL-BREF (World Health Organization Quality of Life Scale – Abbreviated version). Using the EQ-5D visual analogue scale (VAS) of rating of health as a dependent variable, the five EQ-5D and four selected WHOQOL-BREF items were entered as dummy variables in multiple regression analysis.

**Results:**

The additional domains increased the explanatory power of the model from 52 % (EQ-5D only) to 57 % (all domains). The coefficients of Self-Care and Usual Activities were not significant in any model. The most parsimonious model included the EQ-5D domains of Mobility, Pain/Discomfort, Anxiety/Depression, Concentration, and Sleep (adjusted r^2^ = .57).

**Conclusions:**

The EQ-5D-3L performed well, but the addition of domains such as Concentration and Sleep increased the explanatory power. The user needs to weigh the advantage of using the EQ-5D, which allows for the calculation of a single summary index, against the use of a set of domains that are likely to be more responsive to differences in HRQoL within community living respondents. The poor predictive power of the Self-Care and Usual Activities domains within this context needs to be further examined.

## Introduction

Health-related quality of life (HRQoL) is a commonly utilized measure to monitor the perceived health status of populations and patients and as an outcome measure in many clinical trials. It is a broad multidimensional concept that relies on self or proxy report of certain aspects of functioning and health, which are regarded as being important to leading a healthy, quality life. There is an increasing interest in the determination of the health of general populations who do not belong to a specific patient group [1–3] and consequently a need to establish the most appropriate set of domains with which to monitor their health state.

There are several generic measures of HRQoL in common use including the EQ-5D-3L [2], the WHOQOL-BREF (World Health Organization Quality of Life Scale – Abbreviated form) [4], and the SF (Short Form Health Questionnaire) series [5]. The number of items varies from five in the EQ-5D-3L to 36 in the SF-36 questionnaire. All of the above instruments have been proven valid and reliable in many contexts, across many health conditions, and for varied purposes. There are common domains in most of these instruments. The WHOQOL-BREF consists of 26 questions analyzed under the four domains of physical, psychological, social, emotional, and environmental, whereas the SF-6D (Short Form 6 dimensions) includes 11 items across the six dimensions of physical functioning, role limitations, social functioning, pain, mental health, and vitality [6]. The EQ-5D-3L, in contrast, consists of a single question in each of five domains: Mobility, Self-Care, Usual activity, Pain/Discomfort, and Anxiety/Depression.

The EQ-5D-3L was developed by the EuroQol Group and is a standardized generic instrument for describing and valuing HRQoL [7]. It is widely used and has been translated into many languages, a process that is monitored by the EuroQol Group. The EQ-5D-3L has been shown to have good reliability and validity in the context of the current study, an under-resourced area in which predominantly isiXhosa-speaking people reside [8]. The questionnaire has been used in Southern Africa and in research on quality of life and disability [9]. It is simple to administer and, when piloted in this context, took less than five minutes to complete. It consists of the five descriptor domains listed above and a visual analogue scale (VAS), which allows the respondent to judge their current health status on a scale of ranging from 0 (worst health state imaginable) to 100 (best health state imaginable). An EQ-5D-3L health state may be converted to a single summary index through the use of specific algorithms developed through extensive preference-based valuation studies in many countries [10]. The difference between the summary index and the VAS score has been described as follows: “the health status index is based on a set of weights derived from values from general population samples, … in contrast to the respondent’s or patient’s own assessment of his/her health state (EQ VAS scores)” (p.11) [11]. The VAS has been used in several studies as the “gold standard” with which to determine the contribution of different demographic and functional domains on the self-reported HRQoL of individuals [12–14].

Although found to perform well in comparison with other HRQoL measures [15] [3], the question has arisen as to whether the EQ-5D-3L is adequately responsive to changes in terms of the number of domains and the number of levels. In response to the latter issue, the EQ-5D-5L, which has five response levels, has been developed and is now in use [16, 17]. However, there is still debate as to whether the five domains of the EQ-5D-3L capture most or all of the elements relevant to HRQoL [18, 19]. One way to explore this is to establish whether the EQ-5D-3L domains predict the self-perceived HRQoL of individuals as reported by the VAS. The difference in scores across respondents may be accounted for by many factors, including demographic, environmental, and health condition-related factors, each of which may result in a change in the mean score of the individual and a corresponding change in the variance of the scores of the respondents. The question is whether the five domains account for a significant proportion of the variance, that is the difference in scores between people, or are there alternate and additional domains which would discriminate better between people in different health states?

Although other studies have examined the addition of domains in clinical populations such as those related to skin irritation and self-confidence in psoriasis [20], sleep [19] or cognition in dementia [18], there has been no study to investigate which domains actually discriminate between the responses of those with problems within the community. Health technology assessment requires the development of value sets, but time-trade-off methods such as that used in the Yang et al. study [19] do not necessarily assist the researcher in determining which health states should be valued. For example, even if there is general consensus that having severe problems in a specific domain detracts the most from HRQoL, this is of little relevance if no one within the population of interest reports this health state. In other words, it is our contention that time trade off (TTO) and other valuation techniques are less useful in determining which domains should be included than studies on the actual explanatory power of these domains within the studied population.

Whereas it might appear logical to include as many aspects of self-perceived functioning as possible in a HRQoL instrument, this increases the length of the questionnaire and results in a greater investment of time and resources to gain information. Longer questionnaires may be appropriate in the clinical setting, but shorter questionnaires are less expensive to use for population-based studies. In addition to the increased time commitment, questionnaires that allow for comparison between different countries and different language groups need to undergo rigorous translation. The longer the questionnaire the more complex and more expensive the translation process becomes. In addition, there has been a great deal invested into the valuation of the EQ-5D-3L to develop the utility index, and a longer instrument would require additional and more complex valuation exercises, as the computation of utility indices becomes more technically challenging as more domains are added [12]. The addition of further domains to the EQ-5D-3L is not a trivial matter as it may well alter the value of currently utilized coefficients. The paper by Yang et al. [19] on the impact of adding a sleep dimension to the EQ-5D-3L provides evidence of nonadditivity of dimensions. They state that if the relationship between dimensions of health is not additive it would be problematic to have a core preferences value set of a preference-based measures (such as EQ-5D-3L) and then to simply add relevant dimensions and their associated decrements on to it.

It therefore behooves the developers of generic HRQoL measures to produce instruments that include all the important aspects of HRQoL but not to include redundant questions. The EQ-5D-3L clearly has the most parsimonious set of items. It is one of the most widely used descriptive systems [21] and is the instrument of choice of the National Institute of Health and Clinical Excellence (NICE) in the United Kingdom [19]. The purpose of this study was to see whether the ability of the EQ-5D-3L to discriminate between participants in a large community-based study was enhanced by the addition of certain domains, using a similar methodology to a study testing the addition of five further domains in a Swiss sample [12]. The additional items in the current study were not arbitrarily chosen but drawn from nonoverlapping items in the WHOQOL-BREF, which has been translated and validated in many different contexts. The domains included several of those identified by Whynes [13] and Perneger and Courvoisier [12] as possible candidates for inclusion. The WHOQOL-BREF was used in the community study as it has been found useful in large epidemiological studies [22]. It has been extensively tested and used in low – to middle-income countries within community settings [23–25]. It was therefore chosen to provide information regarding possible additional domains.

The purpose of this study was to establish to what extent the variance in the health of community-based populations could be explained by the EQ-5D domains and what the gain in discriminative ability of the instrument would be if additional items were added. It was not disputed that additional items may detract from HRQoL or that they are not important, but rather whether their addition to the EQ-5D would increase the responsiveness of the instrument by accounting for an increased variance within a community-based sample. A further aim was to establish which set of domains accounted for the most variance.

## Methodology

This paper arose out of a study on the prevalence and impact of disability on HRQoL in people living in one of the least-resourced areas of Cape Town. The EQ-5D-3L-3L and the WHOQOL-BREF were utilized with other instruments to establish the prevalence and impact of disability on functioning and HRQoL. The data presented here were therefore gathered opportunistically and the results represent secondary analysis of the results of the survey.

### Sample

Households were identified using randomised cluster sampling, with clusters being identified in areas including formal housing (brick), informal (tin structures), and backyard dwellings (rented out by home owners). A Google Maps aerial view of the areas was used to enumerate the area for cluster sampling. Three clusters were chosen of each dwelling type, and in each cluster four streets were randomly chosen. Ten dwellings in each street were identified on the map, starting from the second house to the left of the street corner and then including every second house from this point on until 10 houses were visited. If for any reason, respondents in the identified household could not be interviewed, an additional visit at an alternative time was attempted before exclusion. Subjects included all adults over the age of 18 years, who were either the most senior member present at the time of the visit or the head of the household. In addition, household members older than 18 years of age who were identified as having a disability through the use of the Washington Group Screening Questions [26] were included and interviewed at a subsequent visit. Respondents who reported no problems in any of the 10 domains tested were excluded from analysis. As proxy report may not be as valid as self-report [27, 28], respondents who were unable to respond on their own behalf were excluded from the study. A sample size of at least 333 was required to yield 80 % power given an effect size of .05 (small), 10 predictor variables, and a significance level of *p* = .05.

### Instrumentation

Apart from demographic and other questionnaires, the participants filled in the WHOQOL-BREF and the EQ-5D-3L. The Washington Group set of six questions was used to identify those with disabilities. All of these instruments were translated into the vernacular, isiXhosa, following a process of forward and backward translation followed by cognitive debriefing.

### Data analysis

The domains chosen for inclusion in analysis were those deemed not to be included in the constructs already included in the EQ-5D-3L (Table 1). The WHOQOL-BREF items are broader as it purports to measure quality of life and is not restricted to HRQoL. Only those items directly relating to health and functioning were considered for this analysis, and items relating to personal or environmental factors were excluded. Items included were those related to Sleep, Energy, Bodily Appearance, Concentration, and Sexual Activity.

**Table 1:** Comparison of WHOQOL-BREF and EQ-5D-3L domains

WHOQOL-BREF^a^ domain	Item	Equivalent EQ-5D-3L domain
Physical Health	Activities of daily living	Self-Care/ Usual activities
	Dependence on medicinal substance and medical aids	Environmental factor according to ICF^b^
	Energy and fatigue	Not included in EQ-5D-3L
	Mobility	Mobility
	Pain/Discomfort	Pain/Discomfort
	Sleep and rest	Not included in EQ-5D-3L
	Work capacity	Usual activities
Psychological	Bodily image and appearance	Not included In EQ-5D-3L
	Negative/ positive feelings	Anxiety/depression
	Self esteem	Personal factors according to the ICF
	Spirituality	Not health-related
	Thinking, learning, memory, concentration	Not included in EQ-5D-3L
Social relationships	Personal relationships	Not included in EQ-5D-3L
	Social support	Environmental factor according to ICF
	Sexual activity	Not included in EQ-5D-3L
Environmental		Environmental factors according to ICF

^a^World Health Organization Quality of Life Scale – Abbreviated version

^b^International Classification of Functioning, Disability and Health [38]

Descriptive statistics were used to describe the sample. As the WHOQOL-BREF yields ordinal data, Spearman’s correlation was used to explore the relationship between the EQ-5D-3 L VAS and the equivalent generic item in the WHOQOL-BREF “How would you rate your quality of life?” from “Very poor” to “Very good.” Multiple regression analysis was used and the first model used forward stepwise entering of all items. An inclusive model, which included all variables entered simultaneously, was then used. Finally, a model that included the EQ-5D-3L items exclusively was developed. The dependent variable was the EQ-5D-3L VAS score. The WHOQOL-BREF responses were recoded to allow comparison with the EQ-5D-3L. “Very dissatisfied” and “Dissatisfied” were equated with having problems in those domains. Dummy variables were created for each of the five EQ-5D-3L domains and the WHOQOL-BREF questions relating to Concentration, Energy, Body appearance, Sleep, and Sexual activity (one each for “Some problems” and “Severe problems”). Residual analysis identified outliers with scores more than three standard deviations from the mean, and these were excluded before the final analyses were presented (two outliers in the complete analysis and three in the EQ-5D-3L domains only).

### Ethical considerations

The study was approved by the Human Research Ethics Committee of the University of Cape Town. All participants gave informed consent.

## Results

There were 590 participants, of which 79 were people with identified disabilities (PWD) (Table 2). As 14 of these were unable to fill in the questionnaire for themselves, their responses were excluded, which resulted in an effective total of 65 PWD and 511 community members who were not disabled (per Washington Group definition) but might have had health conditions. There were 280 (47.5 %) of the total sample who reported no problems in any of the 10 domains, and these were excluded from analysis, leaving 310 respondents. There were approximately equal numbers of males (52.2 %) and females (47.8 %), and the mean age of the sample was 45.0 years (14.3). Table 2 lists the self-reported causes of disability.

**Table 2:** Diagnoses of those with disability

Underlying cause	Count	Percent
Chronic diseases of lifestyle	23	41.8
Unintentional trauma	16	29.1
Other/missing	18	14.5
Intentional trauma	6	10.9
HIV/Tuberculosis	2	3.6
Total	65	100.0

*N* = 55. Only main underlying cause reported

The responses to the descriptor domains of the EQ-5D-3L indicate that Anxiety/depression was the domain most affected (79.7 % reporting problems), followed by Pain/Discomfort (57.4 % reporting problems). There were few respondents who reported severe problems with either Mobility or Self-Care (Table 3), with only five reporting severe problems with Self-Care.

**Table 3:** Frequencies of responses to EQ-5D-3L domains

	No problem	Some problem	Severe problem	Missing	Total
Mobility	199	105	6		310
%	64.2	33.9	1.9		100.0
Self-Care	262	41	5	1	310
%	86.0	12.8	1.1	.3	100.0
Usual Activity	189	98	22	1	310
%	61.0	31.6	7.1	.3	100.0
Pain/Discomfort	132	140	35	2	310
%	42.6	45.2	11.3	.65	100.0
Anxiety/depression	63	206	40		310
%	20.3	66.5	12.9		100.0

*N* = 310

The WHOQOL-BREF domain in which most problems were experienced was in Sexual activity, with 14.2 % reporting problems with their sexual life. However, there were 42 missing responses to this domain. This was followed by Energy, with 12.3 % with some or severe problems (Table 4).

**Table 4:** Frequency of responses to the WHOQOL-BREF^a^ domains

	Very satisfied	Satisfied	Neither	Dissatisfied	Very dissatisfied	M	Total	With problems
Sleep	49	132	98	26	5	0	310	31
%	25.3	51	17.5	4.3	1.2	0.5	100	5.5
Concentration	76	136	66	24	5	3	310	29
%	24.5	43.9	21.3	7.7	1.6	1	100	9.3
Energy	70	131	71	31	7	7	310	38
%	22.6	42.3	22.9	10	2.3	0.7	100	12.3
Sex life	96	92	36	26	18	42	310	44
%	31	29.7	11.6	8.4	5.8	13.5	100	14.2
Body appearance	39	169	77	20	11	0	310	31
%	12.9	52.6	24.8	6.5	3.5	0	100	10

^a^World Health Organization Quality of Life Scale – Abbreviated version

*N* = 310

**Fig. 1:**
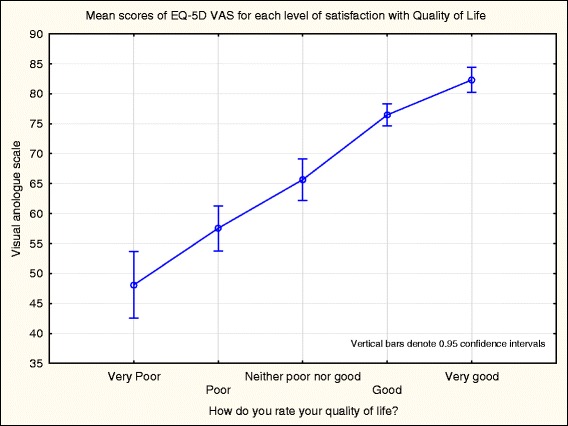
The mean EQ-5D-3L VAS scores for each level of the WHOQOL-BREF item “Level of satisfaction with Quality of Life”

The mean VAS of the EQ-5D-3L, which ranges from 0 (worst health imaginable) to 100 (best health imaginable), was 66.8 (SD = 20.8). There was a significant correlation between the VAS of the EQ-5D-3L and the WHOQOL-BREF item “How would you rate your quality of life?” (Spearman- R = .64, *p* < .001), and a one-way ANOVA indicated that there was a significant effect of level of satisfaction with health (WHOQOL-BREF item) and mean score on the VAS (Fig. 1, F(4, 305) = 83.9, *p* < .001), with a post-hoc Tukey test indicating that the VAS of each level was significantly different to each other level.

The results of all the models developed indicated that, with the exception of Severe Self-Care, the items were logically consistent. The “Severe” level resulted in a greater decrement in the VAS than the “Some” level and every item decreased the perceived health state. Forward stepwise multiple regression analysis was done first to establish which items contributed most to the increase in adjusted r^2^. The first two items to be entered were from the EQ-5D-3L and were some problems with Mobility and Severe Pain or Discomfort, which together accounted for 43 % of the variance. The next two items were the two levels of problems with Concentration from the WHOQO-BREF, and these added 8 %. Some Pain/Discomfort and the two Anxiety/Depression items from the EQ-5D-3L then added a further 5 %.

The results of the regression analysis with all EQ-5D-3L and WHOQOL-BREF domains included are presented in Table 6. Severe problems with concentration resulted in the greatest deficit, −21.4. The model accounted for 57 % of the variance.

Another model (Table 7) was developed, which included a parsimonious set of domains, of which at least one level (Some or Severe problems) added more than 1 % to the explained variance (Table 5). As the coefficient of Severe problems with Sleep was large (−16.5) and significant (*p* = .019, Table 6), Sleep was included in the parsimonious model. This model, which included three of the EQ-5D-3L domains and two of the WHOQOL-BREF domains, accounted for 57 % of the variance (Table 7).

**Table 5:** Forward stepwise multiple regression steps to enter

	Step + in/-out	Multiple R-square	R-square change	F - to entr/rem	*p*-value	Origin
Mobility Some	1	0.30	0.30	129	0.000	EQ-5D-3L
Pain/Discomfort Severe	2	0.43	0.13	72	0.000	EQ-5D-3L
Concentration Severe	3	0.47	0.04	22	0.000	WHOQOL^a^
Concentration Some	4	0.51	0.04	24	0.000	WHOQOL
Pain/Discomfort Some	5	0.53	0.02	12	0.001	EQ-5D-3L
Anxiety/Depression Severe	6	0.54	0.02	11	0.001	EQ-5D-3L
Anxiety/Depression Some	7	0.55	0.01	7	0.007	EQ-5D-3L
Bodily Appearance Some	8	0.56	0.01	7	0.011	WHOQOL^a^
Sleep Severe	9	0.57	0.01	5	0.021	WHOQOL^a^
Energy Some	10	0.58	0.01	5	0.029	WHOQOL^a^
Mobility Severe	11	0.59	0.01	6	0.016	EQ-5D-3L
Sex Life Some	12	0.59	0.00	3	0.090	WHOQOL^a^
Sex Life Severe	13	0.59	0.00	2	0.204	WHOQOL^a^
Usual Activities Some	14	0.60	0.00	1	0.226	EQ-5D-3L

*N* = 308, two outliers removed

^a^World Health Organization Quality of Life Scale – Abbreviated version

**Table 6:** Results of multiple regression analysis of EQ-5D-3L plus all other domains

	b	Std.Err. of b	t(287)	*p*-value
Intercept	86.0	2.1	41.5	0.000
Mobility Some	−13.5	2.4	−5.7	0.000
Mobility Severe	−17.4	8.6	−2.0	0.043
Self-Care Some	−0.9	2.8	−0.3	0.753
Self-Care Severe	8.8	10.2	0.9	0.386
Usual Activities Some	−3.3	2.5	−1.3	0.193
Usual Activities Severe	−3.7	4.5	−0.8	0.406
Pain/Discomfort Some	−4.5	2.0	−2.2	0.028
Pain/Discomfort Severe	−14.0	4.0	−3.5	0.000
Anxiety/Depression Some	−6.3	2.0	−3.1	0.002
Anxiety/Depression Severe	−12.8	3.1	−4.2	0.000
Concentration Some	−8.8	3.5	−2.5	0.013
Concentration Severe	−20.9	6.9	−3.0	0.003
Energy Some	−6.7	3.3	−2.0	0.043
Energy Severe	−4.8	7.4	−0.6	0.517
Sleep Some	0.1	3.7	0.0	0.968
Sleep Severe	−15.1	8.0	−1.9	0.061
Sex Life Some	−4.7	2.9	−1.6	0.107
Sex Life Severe	−5.6	3.9	−1.4	0.153
Bodily Appearance Some	−7.0	3.5	−2.0	0.045
Bodily Appearance Severe	1.6	5.2	0.3	0.760

*N* = 308, two outliers excluded

Adj r^2^ = .57

**Table 7:** Results of model including a parsimonious set of domains (*N* = 307)

	Unstandardized b	Std.Err. of b	t(296)	*p*-value
Intercept	84.9	2.032	41.81	0.000
Mobility Some	−16.7	2.039	−8.17	0.000
Mobility Severe	−15.5	6.097	−2.54	0.011
Pain/Discomfort Some	−5.8	1.867	−3.09	0.002
Pain/Discomfort Severe	−15.9	3.702	−4.30	0.000
Anxiety/Depression Some	−5.7	1.978	−2.86	0.005
Anxiety/Depression Severe	−12.7	2.985	−4.24	0.000
Concentration Some	−10.7	3.253	−3.28	0.001
Concentration Severe	−28.1	6.416	−4.38	0.000
Sleep Some	−3.3	3.230	−1.03	0.306
Sleep Severe	−16.8	7.114	−2.37	0.019

*N* = 307, three outliers excluded

Adj r ^2^ = .57

The final model included only the EQ-5D-3L domains, and this accounted for 52 % of the variance, once three outliers were excluded. In this model (Table 8), Severe problems with Mobility and Pain/Discomfort detracted most from HRQoL, followed by Severe Anxiety/Depression. Once again, the Self-Care and Usual Activities domains did not give rise to significant coefficients.

**Table 8:** Regression model for EQ-5D-3L – exclusive model, only EQ-5D-3L domains

	b	Std.Err. of b	t(296)	*p*-value
Intercept	84.7	2.14	39.69	0.000
Mobility Some	−14.8	2.43	−6.08	0.000
Mobility Severe	−20.4	8.71	−2.34	0.020
Self-Care Some	−2.1	2.91	−0.72	0.471
Self-Care Severe	−6.2	10.25	−0.60	0.548
Usual Activities Some	−2.7	2.60	−1.03	0.302
Usual Activities Severe	−1.6	4.63	−0.36	0.722
Pain/Discomfort Some	−5.9	2.11	−2.80	0.005
Pain/Discomfort Severe	−20.7	3.79	−5.45	0.000
Anxiety/Depression Some	−5.9	2.10	−2.82	0.005
Anxiety/Depression Severe	−12.6	3.15	−4.01	0.000

Three outliers excluded

Adj r^2^ = .52

The change in adjusted r^2^ is summarized in Table 9.

**Table 9:** Summary of the different models

Model	Items	Adjusted r^2^
Inclusive	All EQ-5D-3L domains, Concentration, Bodily Appearance, Sexual Activity, Energy, Sleep	.57
Parsimonious model	Mobility, Pain/Discomfort, Anxiety/Depression, Concentration, Sleep	.57
EQ-5D-3L	EQ-5D-3L domains	.52

Plots of residuals against predicted values indicated no evidence of heteroscedasticity in the models, as the spread of residuals was even across the values of the predicted VAS. In no model was the variable inflation factor (VIF) greater than 2.9 and the tolerance values were all larger than .35, which indicated that there was no evidence of multicollinearity in any model.

## Discussion

We set out to explore the impact of adding additional domains to an established HRQoL instrument, the EQ-5D. As it has performed well in other community-based studies in South Africa [29, 30], it was anticipated that the existing domains would be sufficient and that additional domains would not be needed. The results partially supported this assumption, as the EQ-5D exclusive model accounted for only 5 % less variance than models including other domains (52 %). However, the coefficients of the domains of Self-Care and Usual Activities were nonsignificant. A parsimonious model including three EQ-5D and two WHOQOL-BREF domains was the most efficient in discriminating between respondents (57 % of the variance).

The responses to the instruments were consistent (apart from Severe problems with Self-Care) and the association between the two forms of HRQoL measures (the VAS from the EQ-5D-3L and the rating of quality of life from the WHOQOL-BREF questionnaires) supports the validity of the utilization of the EQ-5D-3L and the WHOQOL-BREF within the context of the study. Internal validity is further strengthened by the significant difference in VAS across the different levels of the WHOQOL-BREF General Satisfaction with Life item. All coefficients were negative, and there was overall logical consistency of responses with respect to level, in that the Severe detracted more than the Some level in almost every case (apart from Severe problems with Self-Care). In addition, there was no evidence of heteroscedasticity or collinearity in the models, which therefore met the assumptions necessary for regression analysis. Only two or three outliers were excluded in each model, which implies that the vast majority of participants responded appropriately to the questionnaires.

It is clear that all models performed well and accounted for more than 52 % of the variance, which was acceptable, in comparison with other studies using the VAS as an outcome measure. The adjusted r^2^ values were higher than another study that used the VAS as dependent variable and the domains as predictors (46 % in a general resident Swiss population [12]). However, the current study excluded those with no problems in any domain, whereas the Swiss study did not. It might be expected that in a sample of people with a specific condition, an outcome-specific instrument may have better predictive ability than in a community-based sample, but this was not the case. A study using the valuation score of the EQ-5D-3L as the outcome variable and scores on an arthritis-specific measure as predictors accounted for a comparable 56 % of the variance, which the authors maintain is at the high end of the range (31–66 %) for published models [31]. Similarly, in a study on different patient populations, the variance accounted for was 47 % using only the EQ-5D-3L domains, rising to 56 % with the addition of five additional domains [12]. It can be concluded that all three models performed well and accounted for a similar amount of variance.

How important is a difference in 5 % of the variance? Swinburne et al. [20] found that the addition of two domains to the EQ-5D-3L increased the explanatory power with regard to psoriasis-specific instruments by approximately 23 %, a much larger increment than found in this study. They concluded that the regression analysis showed that the new measure was “much better at predicting psoriasis outcomes when compared with the unmodified EQ-5D-3L questionnaire.” Whynes [13] in investigating the addition of five more domains to those of the EQ-5D-3L found an increase from 47 to 56 %, which he considered to be a “substantial improvement” [14], although he admits that it may not be sufficient. In contrast, the Yang study on the addition of a Sleep domain reported an increase from 28 to 34 % in TTO explanatory power resulting from the addition of a Sleep dimension. Their study concluded that, based on other parameters, Sleep did not make a significant impact on the values people place on the EQ-5D-3L. There would seem to be no consensus as to what constitutes a meaningful change in the adjusted r ^2^. We suggest that the impact of a 5 % difference would depend on the nature of the study and the questions to be answered. If the difference to be evaluated between the mean values of the VAS between community-based groups was anticipated to be small, then it would make a difference to the outcome, and the alternate set of domains should be used. On the other hand, if a large difference is anticipated (e.g., between those living with HIV in the community and those without), then the difference in explanatory power might not be important.

These findings are inconsistent with other studies that examined the performance of the EQ-5D-3L plus different “bolt-on” domains. A recent study on the inclusion of a sleep domain concluded that there was no apparent benefit to adding a Sleep dimension to the EQ-5D-3L but that further research is needed to determine how additional dimensions could be identified and tested [19]. Similarly, research on the addition of a cognitive domain, such as concentration, concluded that the EQ-5D-3L performed well within a population with cognitive impairments and that the adjustment of the current classification system by adding a cognitive dimension was suggested to be unnecessary [18]. On the other hand, there are studies that have recommended the inclusion of other domains in HRQoL instruments. Using regression analysis on a large sample of patients with diverse conditions, a generic core set was developed, which included the body functions of Energy and drive, Emotional functions and Pain. The activities identified included carrying out a daily routine, walking, moving around, and the participation function of remunerative employment [32]. These items are remarkably similar to those in the EQ-5D-3L despite having been developed through a very different empirical process of patient assessment. It was noted that Energy and drive functions were highly correlated with Emotional functions, and it might be for this reason that the Energy domain did not add to the explanatory power of the EQ-5D-3L [32]. A population-based study to establish health dimensions that should be included in multiattribute health utility assessment included the EQ-5D domains, the domains explored in the current study, with Contact with Others and Seeing as additional domains. They concluded that the additional domains added 9 % to the explained variance and suggested that the addition of further items should be explored in the future [12].

The responses to the EQ-5D-3L domains were similar to other studies in under-resourced areas in Cape Town [8, 33] and elsewhere, in that the Self-Care domain was reported to be the least affected and the Pain/Discomfort and Anxiety/Depression domains were the most affected [1]. The VAS of 66.8 was less than that reported in a study reporting the validation of the EQ-5D-3L in a similar population in Cape Town (80.1, SD = 20.4). However, only those with problems in at least one domain were included in this study and the previous survey was done several years ago (2003), before the economic recession [1]. The frequencies of those reporting problems in the selected WHOQOL-BREF domains were considerably lower than in other studies. A study that administered the WHOQOL-BREF to adult community residents in a major metropolitan city in southern China, for example, reported approximately 16 % of respondents had problems in the domains of Energy, Concentration, and Body image, compared to the 5.5–14.2 % seen in the current study. Relatively, the South African population had more problems with Sexual activity, 14.2 % compared to 5 % in the Chinese group [23], although there were a large number of missing responses to this question. It is unclear why there is such a discrepancy.

The nonsignificant predictive coefficients of some of the EQ-5D-3L domains also need discussion. Although there were those with disabilities within the current sample, there were very few respondents with problems in the domain of Self-Care. The small number may have contributed to the lack of contribution to the variance by this domain in the Inclusive model and the counterintuitive regression coefficient. In his study on the reported VAS of patients with different health conditions, Whynes (2013) similarly found neither of the coefficients for the level 2 and the level 3 variable in the Self-Care domain achieved statistical significance [13]. His sample did not include those with severe illness, and the lack of significance in this domain may not be the case in those who are very ill or severely disabled. Self-Care was found to contribute significantly to decreased HRQoL in patients after back surgery [34] and in the later stages of dementia [18]. It is more difficult to explain the nonsignificant coefficients of the Usual Activity levels, which is in contrast to findings within clinical populations, e.g., with dementia [18]. Although not reported in the Results section, we did find that once the domain of Mobility was excluded from analysis, the coefficients for both levels of Usual Activities became significant. The two domains may be tapping into a similar construct within a community-based population.

It is noteworthy that, apart from Mobility and Pain/Discomfort, the EQ-5D-3L domains were not the domains which had the largest coefficients in the inclusive model. Clearly the five additional domains were important to this group of subjects. This is maybe not surprising as the level of HIV infection stands at 6.26 % in the Western Cape [35], and the domains of Concentration and Sleep have been found to be those most affected by those infected with the virus [36]. Although few PWD reported having HIV, it is likely to have been prevalent in the general sample.

A limitation of the study is that that the phrasing of the WHOQOL-BREF and the EQ-5D-3L items is different. The WHOQOL-BREF asks for a rating of satisfaction for some items (e.g., Sexual activity and Body appearance) and a rating of how well the function is performed in others (such as Concentration). The EQ-5D-3L, on the other hand, requires a rating of the degree of problems experienced with each domain. It is difficult to know how this affects the results of the study. In regression analysis, very different variables may be entered, but in order to ensure that the responses from the two questionnaires were comparable, the construct of reporting a problem or not reporting a problem was used across all domains. A respondent may be satisfied with a function but still not be able to do it well. This response would not be identified as being a problem and the impact of using the different phrasing might lead to an under-representation of problems in the WHOQOL-BREF items.

The exclusion from analysis of those reporting no problems in any domains resulted in the development of better models, but it might limit the generalizability of the results to that section of the community who do perceive themselves as having some problems in the domains. However, the inclusion of those with no problems would have added no additional explanatory power. A related limitation is that there were large numbers of respondents who did not answer the question relating to Sexual Activity. This might have biased the results, as the nonresponders might well have been those who had the most problems in this domain. The inclusion of a domain relating to sexual functioning should be reexamined in other cultural contexts in which there is less discomfort in reporting on sexual activity.

Another limitation is that as the study was nested in a larger survey, the order of presentation of the questionnaires was not randomized. Those with disabilities answered the WHOQOL-BREF first and the other respondents the EQ-5D-3L. This may have led to an ordering effect.

Another contentious issue is the use of the VAS as a cardinal rather than an ordinal scale. Some authors have used the VAS as an independent variable in logistic regression by categorizing the scores into poor, fair, and good health [12]. However, other researchers have applied parametric statistics in analysis of VAS outcomes [13, 14, 37], and it appeared to function well in the context of this study. It is generally preferable to use the cardinal values for the different health states elicited through valuation exercises, but this was of course not possible as the domains scores are used to calculate the index score. Another issue is that, as with the study by Peneger et al. [12], we explored the contribution of different domains of functioning to self-perceived health and not the value that society attaches to health states. The use of the VAS as outcome, rather than an outcome generated through a valuation method, such as Time Trade Off as used by Yang et al. [19], might well have resulted in very different models. However, the study set out to determine what problems, as they exist in the community, are predictive of poor HRQoL scores, in order to determine which set of domains is most useful in this context. Valuation exercises are, by their nature, dependent on people valuing hypothetical health states, and this does not give information as to the validity of any particular set of domains within a community.

Finally, it needs to be emphasized that the sample was drawn from a randomly sampled community-dwelling population. The results may well be different if a specific functional limitation was tested within a specific patient group. These conclusions are therefore limited to general population measurement.

## Conclusion

There are obviously several factors that guide the choice of suitable instruments to measure HRQoL, and the investigator needs to weigh the relative advantages of each measure. This study found that a parsimonious set of domains drawn from the two instruments resulted in greater explanatory power, but this was not much greater than that of the EQ-5D-3L domains alone. It is recommended that if the HRQoL of community-based, as opposed to clinical, populations is to be examined, researchers balance the advantage of having an instrument that has preference weights attached against the loss of precision and explanatory power. As the EQ-5D-3L in its present form is widely utilized, and as value sets have been developed in many different languages and for different cultures, there is a good case for continuing with the existing set of five domains. The addition of further domains to the EQ-5D-3L will increase the response burden and will require further translations into over 100 languages and large-scale valuation exercises to determine the utility weights of the expanded instrument. At present there are 243 possible health states, and the addition of a single domain would increase the number to be valued to over 1400. In the light of the relatively small benefit of the additional domains, further research would be required to justify any such additions. It is clear that by their very nature, generic measures of HRQoL will include some domains that are irrelevant and exclude some that are useful, within certain groups of respondents.

However, if no tariffs are required, it is suggested that the parsimonious model be utilized, possibly including a domain related to sexual activity (depending on the cultural context). The additional domains, which should be considered as candidates for bolt-on dimensions, are Concentration, Sleep, and possibly Sexual Activity. The EQ-5D-3L domains of Self-Care and Usual Activities should be excluded within this context.

It should be noted that these conclusions and recommendations cannot be extrapolated to clinical populations in which the patterns of problems reported may be very different.
